# The transcription factor MdWRKY9 is involved in jasmonic acid-mediated salt stress tolerance in apple

**DOI:** 10.1093/hr/uhaf068

**Published:** 2025-03-04

**Authors:** Jiahao Zhao, Shuhui Zhang, Zhicheng Yu, Tingting Gu, Jie Zhang, Lingyu Meng, Zijing Chen, Zongying Zhang, Nan Wang, Xuesen Chen, Wenjun Liu

**Affiliations:** College of Horticulture Science and Engineering, Shandong Agricultural University, Taian 271018, Shandong, China; Collaborative Innovation Center of Fruit & Vegetable Quality and Efficient Production, Taian 271018, Shandong, China; College of Horticulture, Northwest A&F University, Yangling, Shaanxi 712100, China; College of Horticulture Science and Engineering, Shandong Agricultural University, Taian 271018, Shandong, China; Collaborative Innovation Center of Fruit & Vegetable Quality and Efficient Production, Taian 271018, Shandong, China; College of Agricultural Sciences and Technology, Shandong Agriculture And Engineering University, Jinan 250100, Shandong, China; College of Horticulture Science and Engineering, Shandong Agricultural University, Taian 271018, Shandong, China; Collaborative Innovation Center of Fruit & Vegetable Quality and Efficient Production, Taian 271018, Shandong, China; College of Horticulture Science and Engineering, Shandong Agricultural University, Taian 271018, Shandong, China; Collaborative Innovation Center of Fruit & Vegetable Quality and Efficient Production, Taian 271018, Shandong, China; College of Horticulture Science and Engineering, Shandong Agricultural University, Taian 271018, Shandong, China; Collaborative Innovation Center of Fruit & Vegetable Quality and Efficient Production, Taian 271018, Shandong, China; College of Horticulture Science and Engineering, Shandong Agricultural University, Taian 271018, Shandong, China; Collaborative Innovation Center of Fruit & Vegetable Quality and Efficient Production, Taian 271018, Shandong, China; College of Horticulture Science and Engineering, Shandong Agricultural University, Taian 271018, Shandong, China; Collaborative Innovation Center of Fruit & Vegetable Quality and Efficient Production, Taian 271018, Shandong, China; College of Horticulture Science and Engineering, Shandong Agricultural University, Taian 271018, Shandong, China; Collaborative Innovation Center of Fruit & Vegetable Quality and Efficient Production, Taian 271018, Shandong, China; College of Horticulture Science and Engineering, Shandong Agricultural University, Taian 271018, Shandong, China; Collaborative Innovation Center of Fruit & Vegetable Quality and Efficient Production, Taian 271018, Shandong, China

## Abstract

Salt stress is an important abiotic stress affecting the growth and fruit quality of apple fruits. Although jasmonic acid (JA) hormones and WRKY transcription factors (TFs) have both been reported to be involved in plant salt stress responses, the molecular mechanisms by which JA-mediated WRKY TFs regulate salt stress in apples remain unclear. Here, we report the identification of a WRKY family TF from apple, MdWRKY9, and its involvement in apple salt tolerance by regulating the expression of Na^+^/H^+^ antiporters, *MdNHX1*, and *MdSOS2*. Furthermore, we show that the protein repressors MdJAZ5 and MdJAZ10 in the JA signaling pathway can both interact with MdWRKY9 to form a complex and inhibit its DNA-binding and transcriptional activation activity. The JA signal triggers the degradation of MdJAZ5 and MdJAZ10 proteins by the 26S proteasome, disrupting the JAZ–WRKY protein complex and thereby releasing MdWRKY9 to activate downstream gene expression, promoting salt tolerance in apples. These findings provide important insights into the molecular mechanism of the WRKY TFs in JA-mediated salt tolerance in plants.

## Introduction

Global land salinization is a significant environmental issue affecting agricultural productivity, food security, and ecosystems worldwide [1]. Due to the combined effects of natural factors and human activities, >6% of the world’s total land area has been affected by excessive salinity, and the situation is worsening [2, 3]. Salt stress almost affects all processes of plant growth and development, including seed germination, vegetative growth, and reproductive growth. Salt stress first leads to primary damage such as cell ion balance disruption and osmotic stress [1, 4]. Subsequently, it generates a large amount of reactive oxygen species (ROS), causing secondary damage like oxidative stress [5, 6].

Plants have evolved various precise regulatory mechanisms to respond to salt stress, such as salt overly sensitive (SOS) signaling pathway and hormone signaling pathways [7, 8]. Under salt stress, plants can expel excessive Na^+^ from the cytoplasm to maintain low Na^+^ levels and protect themselves from salt stress damage [7, 9]. This process is mainly carried out by Na^+^/H^+^ antiporters, including the plasma membrane-localized Na^+^/H^+^ antiporter, the most well known of which is salt overly sensitive 1 (SOS1), and the vacuolar membrane-localized Na^+^/H^+^ antiporter, such as NHX1 and NHX2 [10–13]. In plant cells, after SOS3/CBL4 senses the cytoplasmic Ca^2+^ signal triggered by salt stress, it can bind to SOS2/CIPK24 to form a protein complex and phosphorylate it. This process activates the expression of the SOS1, promoting the expulsion of excess Na^+^ from the cell [14, 15]. Additionally, NHX1 and NHX2, which are localized to the vacuolar membrane, are primarily responsible for sequestering Na^+^ in the vacuole [13, 16]. Overexpression of *AtNHX1* in *Arabidopsis*, *Brassica napus*, *Zea mays* L., and *Tritivum aestivum* L. results in significantly higher salt tolerance compared to wild-type (WT) plants [17–20].

In addition to the Ca^2+^-mediated SOS signaling pathway, plant hormones, such as abscisic acid (ABA), brassinosteroids (BR), ethylene (ETH), salicylic acid (SA) and jasmonic acid (JA) etc., also play important roles in salt stress signal transduction and activation of defense responses [21]. Salt stress causes a rapid increase in endogenous ABA levels, which activates SnRK2 protein kinases and phosphorylates downstream effector proteins in response to the stress [22]. BR can increase the activity of Na^+^/H^+^ antiporters, reducing intracellular Na^+^ levels and increasing K^+^ levels, thereby alleviating salt stress [23]. Salicylic acid can prevent salt-induced K^+^ loss, thereby enhancing salt tolerance in *Arabidopsis* [24]. ETH, on the other hand, regulates the response to salt stress by modulating the production and scavenging mechanisms of ROS [25, 26]. Recent research indicates that boosting endogenous JA levels and applying exogenous JA can enhance plant salt tolerance [27]. For instance, salt stress can activate the JA signaling pathway in the elongation zone of *Arabidopsis* roots, inhibiting cell elongation [28]. In tomato, endogenous JA can enhance salt tolerance by maintaining intracellular ROS homeostasis [29]. However, the precise molecular and physiological mechanisms underlying JA’s enhancement of plant salt tolerance remain largely unknown.

Transcription factors (TFs) play a crucial role in regulating gene expression under salt stress by binding to the promoters of functional genes, thereby activating or repressing their transcription, including TF families such as MYB, NAC, bZIP, and WRKY [30–32]. WRKY TFs, one of the largest families in higher plants, significantly influence plant growth and development and play a key role in various salt stress response pathways [33]. WRKY TFs activate downstream genes by binding to the conserved W-box (T)TGAC(C/T) elements in their promoters [34, 35]. Previous studies have shown that WRKY TFs function synergistically or independently in the ABA signaling pathway induced by salt stress [33]. They may also help plants adapt to salt stress by reducing ROS levels and increasing the synthesis of osmotic substances [36]. Additionally, WRKY TFs have been reported to directly bind to the promoters of Na^+^/H^+^ antiporters such as *SOS1*, *SOS2*, and *NHX1*, participating in the plant’s salt stress response by controlling ion homeostasis [37–39]. As of now, there are still many unknowns about the molecular mechanisms by which WRKY TFs respond to JA signals to regulate plant salt tolerance.

Apples (*Malus domestica*), as one of the world’s four major fruits, are widely cultivated worldwide due to their strong ecological adaptability, high nutritional value, and long supply period [40]. As a perennial woody plant, apples are greatly influenced by changes in soil environment in terms of tree growth, development, and fruit quality [41]. Here, we report the identification of a WRKY family TF from apple, MdWRKY9, and its involvement in apple salt tolerance by regulating the expression of Na^+^/H^+^ antiporters, *MdNHX1* and *MdSOS2*. Furthermore, we show that the protein repressors MdJAZ5 and MdJAZ10 in the JA signaling pathway can both interact with MdWRKY9 to form a complex and inhibit its DNA-binding and transcriptional activation activity. The JA signal triggers the degradation of MdJAZ5 and MdJAZ10 proteins by the 26S proteasome, disrupting the JAZ–WRKY protein complex and thereby releasing MdWRKY9 to activate downstream gene expression, promoting salt tolerance in apples. These findings provide important insights into the molecular mechanism of the WRKY TFs in JA-mediated salt tolerance in plants.

## Results

### Salt stress inhibits the growth of apple roots and induces the accumulation of endogenous JA

**Figure 1:**
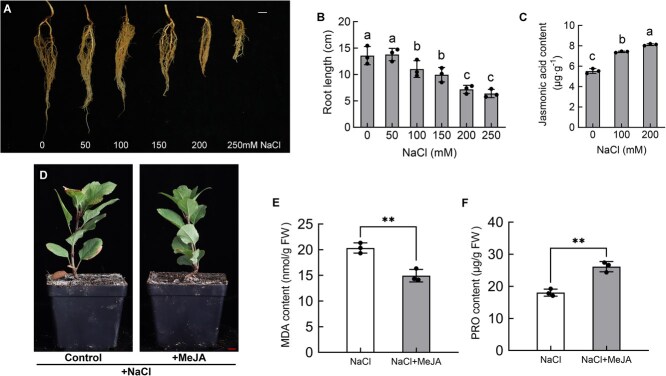
Salt stress inhibits the growth of apple roots and induces the accumulation of endogenous JA. (A) Root growth status of potted *M. hupehensis* Rehd. seedlings after salt stress treatment with NaCl (0, 50, 100, 150, 200, and 250 mM NaCl). Scale bar = 1 cm. (B) Root length of apple seedlings after salt stress treatment with different NaCl concentrations. (C) Endogenous JA content accumulated in apple roots at NaCl concentrations of 0 mM,100 mM, and 200 mM. Values are means ± SD of three independent biological replicates (*n* = 3). Significant differences were determined by one-way ANOVA followed by a Tukey’s test (*P* < 0.05). (D–F) Morphological changes of M9T337 seedlings treated with 150 mM NaCl and 150 mM NaCl combined with 25 μM MeJA. (D) Plant Phenotype, Scale bar = 1 cm, (E) MDA content in the roots, (F) PRO content in the roots.

Plant roots are the first organs to sense changes in the soil environment. The inhibition of root growth when plants are subjected to salt stress is an important adaptive strategy to avoid high salt damage [42]. To explore the effect of salt stress on apple root growth, we used *Malus hupehensis* Rehd. seedlings, which are commonly used as apple rootstocks in production, as test materials and irrigated the potted seedlings with salt solutions of different concentrations (Fig. 1A). The results showed that when the salt concentration reached 100 mM, apple root growth was significantly inhibited, and the inhibitory effect gradually increased with higher salt concentrations (Fig. 1A and B). Additionally, the surface area, total root length, and mean root diameter of apple roots were significantly inhibited when the salt concentration reached 200 mM (Fig. S1). We then selected roots with significant growth inhibition at salt concentrations of 100 and 200 mM and measured their endogenous JA content using high-performance liquid chromatography (HPLC; Fig. S2). The results showed that salt stress significantly induced the accumulation of endogenous JA in apple roots (Fig. 1C). The active form of JA is JA-Ile. Upon examination of its concentration, a notable elevation in the levels of JA-Ile was observed under salt stress conditions (Fig. S3). Collectively, these findings suggest that JA is implicated in the response to salt stress.

To delve into the effects of JA on apple seedlings under salt stress, this study treated apple seedlings with JA under salt stress conditions. Compared to the control group, the degree of salt stress experienced by the JA-treated seedlings was significantly reduced (Fig. 1D). Next, we measured the content of malondialdehyde (MDA) and proline (PRO) in the leaves of apple seedlings (Fig. 1E and F). Compared to the control, the MDA content in the leaves of apple seedlings treated with JA showed a significant decrease, while the PRO content increased significantly. This finding not only robustly confirms the positive effect of JA in mitigating salt stress but also provides valuable insights and a basis for further unraveling its physiological mechanisms.

### MdWRKY9 is significantly induced by salt stress and can improve the salt tolerance of apple callus and Arabidopsis

**Figure 2:**
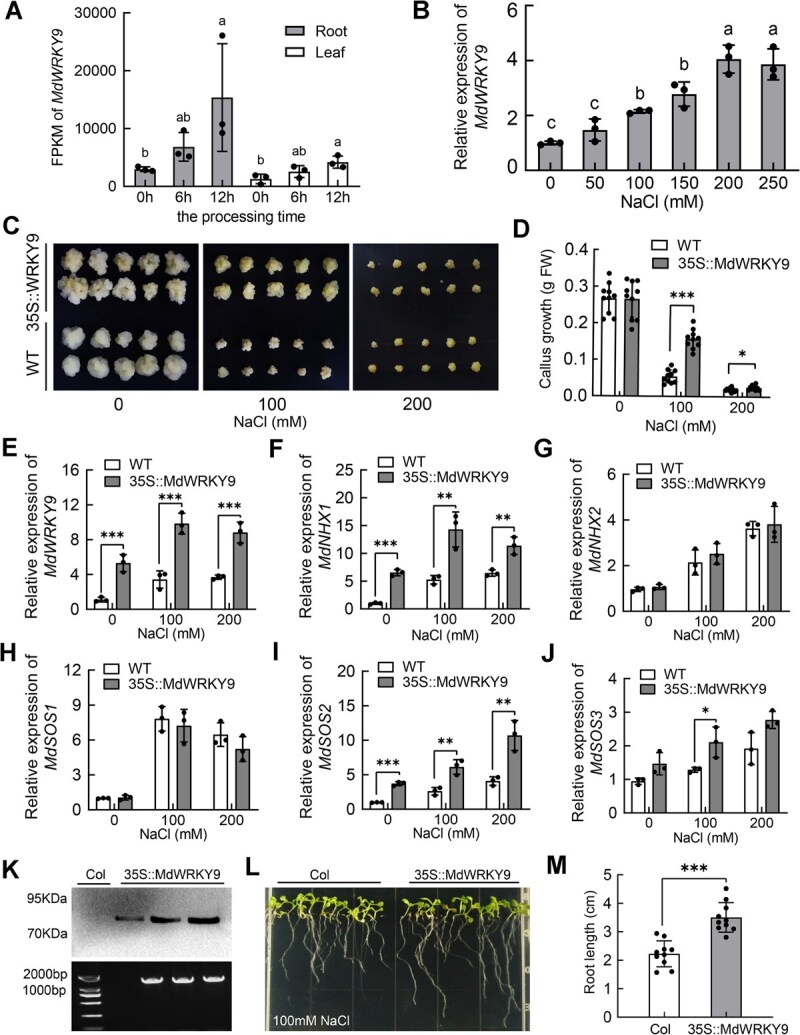
MdWRKY9 is significantly induced by salt stress and improves the salt tolerance of apple callus and Arabidopsis. (A) Previous RNA-sequencing analyses revealed a marked elevation in the FPKM level of *MdWRKY9* specifically in the roots and leaves of apple seedlings subjected to 150 mM NaCl treatment. (B) The study examined the expression patterns of *MdWRKY9* in apple roots in response to varying concentrations of NaCl-induced salt stress, utilizing *MdActin* as a reference gene for normalization. The untreated condition (0 mM NaCl) served as the baseline for comparison. (C) Growth status of WT ‘Orin’ apple callus and *35S::MdWRKY9*-overexpressing callus on MS medium containing 0, 100, and 200 mM NaCl. (D) Growth values of WT ‘Orin’ apple callus and *35S::MdWRKY9*-overexpressing callus on 0, 100, and 200 mM NaCl. FW: Fresh weight. Values are means ± SD of independent biological replicates (*n* = 10). (E–J) The transcript levels of *MdWRKY9* (E), *MdNHX1* (F), *MdNHX2* (G), *MdSOS1* (H), *MdSOS2* (I), and *MdSOS3* (J) in ‘Orin’ apple callus and *35S::MdWRKY9-*overexpressing callus under salt stress treatment with NaCl concentrations of 0, 100, and 200 mM. *MdActin* was used as control. Zero millimolar NaCl treatment of ‘Orin’ apple callus was used as an internal standard. Values are means ± SD of independent biological replicates (*n* = 3). (K) Transgenic *Arabidopsis* seedlings overexpressing *MdWRKY9* were identified using PCR and immunoblotting. (L) Growth status of WT Col-0 and *35S::MdWRKY9*-overexpressing *Arabidopsis* seedlings on MS medium containing 100 mM NaCl. (M) Root length of WT Col-0 *Arabidopsis* seedlings and *35S::MdWRKY9*-overexpressing *Arabidopsis* seedlings on MS medium containing 100 mM NaCl. ^***^P < 0.001, ^**^P < 0.01, ^*^P < 0.05.

In previous studies, we conducted RNA-sequencing (RNA-seq) analyses on the roots and leaves of apple plants under 150-mM salt stress [43]. Here, in-depth exploration of differentially expressed genes revealed a WRKY family TF, *MdWRKY9*, whose expression was significantly upregulated under salt stress in the roots (Fig. 2A, Fig. S4). We then further validated the expression levels of *MdWRKY9* in apple roots under different salt concentrations using Reverse Transcription Quantitative Polymerase Chain Reaction (RT-qPCR). The results were consistent with the RNA-seq, showing that the expression levels of *MdWRKY9* significantly increased with rising salt concentrations (Fig. 2B).

To investigate the role of *MdWRKY9* in salt tolerance, we utilized *Agrobacterium*-mediated stable transformation to enhance the expression of *MdWRKY9* in ‘Orin’ callus, driven by the cauliflower mosaic virus (CaMV) 35S promoter. Transgenic calli with overexpressed *MdWRKY9* (*35S,MdWRKY9*) were then identified using PCR (Fig. S5A) and immunoblotting (Fig. S5B). Compared to WT ‘Orin’ callus, the *35S::MdWRKY9* transgenic callus showed higher transcript levels of *MdWRKY9* and exhibited greater salt tolerance, particularly demonstrating significantly increased growth on plates with a 100 mM salt concentration (Fig. 2C–E). Meanwhile, we also detected the expression levels of the main Na^+^/H^+^ antiporters involved in salt stress in WT and *35S::MdWRKY9* callus, including *MdNHX1*, *MdNHX2*, *MdSOS1*, *MdSOS2*, and *MdSOS3*. The results showed that the expression levels of *MdNHX1* and *MdSOS2* were significantly higher in *35S::MdWRKY9* callus than in WT at both 100 and 200 mM NaCl concentrations (Fig. 2F and I), while the expression of *MdSOS3* was only higher in *35S::MdWRKY9* callus than in WT at 100 mM NaCl concentration (Fig. 2J). Additionally, although the expression levels of *MdNHX2* and *MdSOS1* were induced by salt stress, there was no significant difference between the *35S::MdWRKY9* callus and WT, indicating that their transcription levels were not regulated by *MdWRKY9* (Fig. 2G and H).

To further investigate the salt tolerance function of *MdWRKY9*, we ectopically overexpressed *MdWRKY9* in WT *Arabidopsis thaliana* Col-0. Transgenic *Arabidopsis* seedlings overexpressing *MdWRKY9* were also identified using PCR and immunoblotting (Fig. 2K). The results showed that on the Murashige and Skoog (MS) media supplemented with 100 mM NaCl, *35::MdWRKY9* transgenic *Arabidopsis* plants exhibited better growth, with significantly longer average root lengths compared to the WT control Col-0 (Fig. 2L and M). These results all suggest a positive regulatory role of *MdWRKY9* in salt tolerance.

### MdWRKY9 enhances the transcription of *MdNHX1* and *MdSOS2* by binding to their promoters

**Figure 3:**
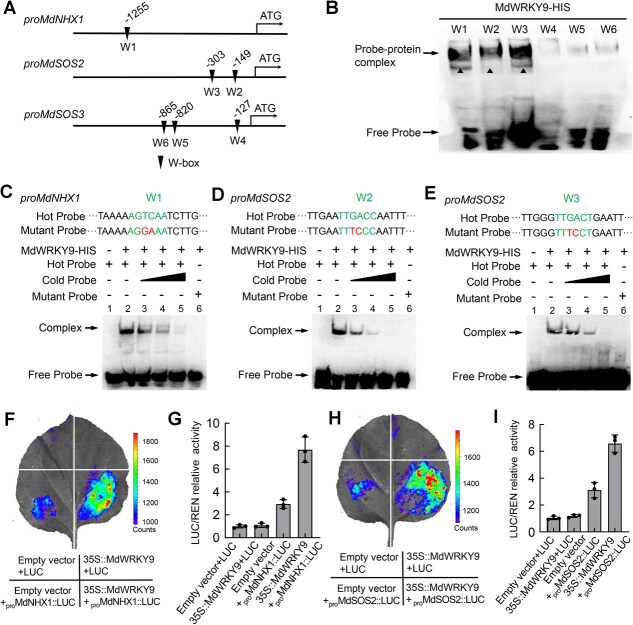
MdWRKY9 enhances the transcription of *MdNHX1* and *MdSOS2* by binding to their promoters. (A) The W-box in the promoters of *MdNHX1*, *MdSOS2*, and *MdSOS3*. Numbers indicate positions of the W-box upstream of their coding sequences. (B) EMSA showing the binding of MdWRKY9 to the W-box *cis*-acting elements in the promoters of *MdNHX1* (W1) and *MdSOS2* (W2 and W3). The black triangles represented that MdWRKY9 combined with the W1, W2, and W3 *cis*-elements. (C–E) The binding of MdWRKY9 to W1, W2, and W3 *cis*-elements was further verified using competition probes and mutation probes. (F–G) A transient LUC reporter assay in tobacco leaves demonstrates the effects of MdWRKY9 on the promoter activity of *MdNHX1*. (H–I) A transient LUC reporter assay in tobacco leaves demonstrates the effects of MdWRKY9 on the promoter activity of *MdSOS2*.

The transgenic results above showed that the expression levels of *MdNHX1*, *MdSOS2*, and *MdSOS3* increased to varying degrees in *35S::MdWRKY9* apple callus, suggesting that they may be regulated by *MdWRKY9* transcription. To verify the regulation of MdWRKY9 on *MdNHX1*, *MdSOS2*, and *MdSOS3*, we first analyzed their promoter sequences and found that *MdNHX1* promoter contains one W-box (W1, −1255), *MdSOS2* promoter contains two W-boxes (W2, −149; W3, −303), and *MdSOS3* promoter contains three W-boxes (W4, −127; W5, −820; W5, −865), respectively (Fig. 3A). We then purified the MdWRKY9 protein fused with an HIS tag, and employed the Electrophoretic Mobility Shift Assay (EMSA) to definitively confirm its binding to the six W-boxes. The results showed that MdWRKY9 binds to the promoters of *MdNHX1* (W1) and *MdSOS2* (W2 and W3), and not to any W-box in the *MdSOS3* promoter (Fig. 3B). Furthermore, addition of unlabeled probes weakened the binding of MdWRKY9, while using mutant probes containing two mutated nucleotides completely abolished this binding (Fig. 3C–E). These results demonstrate that MdWRKY9 can directly bind to the W-boxes in the promoters of both *MdNHX1* and *MdSOS2*.

Subsequently, we used a transient luciferase (LUC) assay in *Nicotiana benthamiana* leaves to investigate the effect of MdWRKY9 on the promoter activity of *MdNHX1* and *MdSOS2*. The coding sequence (CDS) of *MdWRKY9* was fused with the pGreenII 62-SK plasmid as an effector, while the promoter sequences of *MdNHX1* and *MdSOS2* were inserted into the pGreenII 0800-LUC plasmid and fused with the LUC reporter gene, respectively. Luminescence detection showed that coexpression of *35S::MdWRKY9* with *proMdNHX1::LUC* produced significantly stronger fluorescence intensity compared to *proMdNHX1::LUC* combined with the empty vector control (Fig. 3F and G). Similar results were obtained for the promoter activity of *proMdSOS2::LUC* (Fig. 3H and I). We employed Chromatin immunoprecipitation-quantitative real-time PCR (ChIP-qPCR) assay to investigate the enrichment levels of the promoter regions of *MdNHX1* and *MdSOS2*, which contain w-box motifs. The DNA immunoprecipitated with Green Fluorescent Protein (GFP) antibody was compared to that with a nonspecific immunoglobulin G (IgG) antibody control. We found that MdWRKY9 significantly enhanced the enrichment of the *MdNHX1* promoter containing the W1 box sequence in ChIP samples, and similarly observed a significant increase in the enrichment of the *MdSOS2* promoter regions containing W2 box and W3 box in ChIP samples (Fig. S6). These results indicate that MdWRKY9 can enhance the transcriptional activity of *MdNHX1* and *MdSOS2* by binding to their promoters, thereby regulating ion homeostasis under salt stress.

### MdWRKY9 physically interacts with JAZ proteins MdJAZ5 and MdJAZ10 *in vivo* and *in vitro*

**Figure 4:**
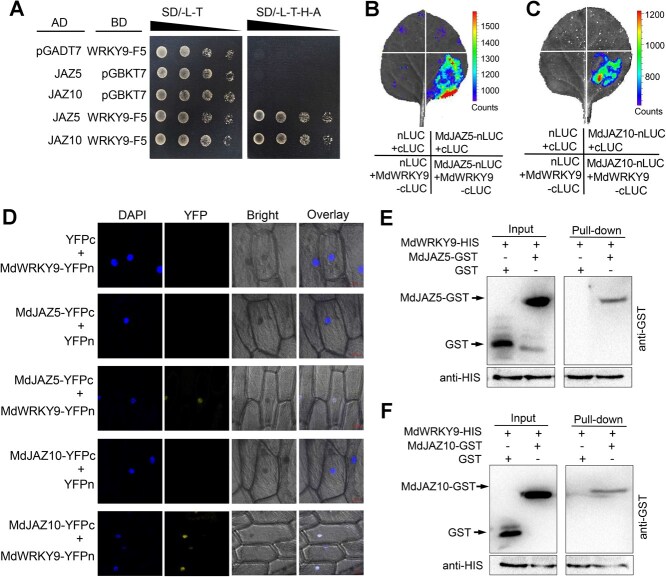
MdWRKY9 interacts with MdJAZ5 and MdJAZ10 *in vitro* and *in vivo*. (A) Y2H assays showing the interaction of MdWRKY9 with MdJAZ5 and MdJAZ10. Each colony was dissolved in 10 μl of 0.9% NaCl and then diluted to 10^−1^ ~ 10^−3^. (B–C) LCI assays showing the interaction of MdWRKY9 with MdJAZ5 (B) and MdJAZ10 (C). (D) BiFC assay showing the interaction of MdWRKY9 with MdJAZ5 and MdJAZ10. DAPI staining was used to visualize the nuclei; Scale bar = 50 μm. (E–F) Pull-down assay showing the interaction of MdWRKY9 with MdJAZ5 (E) and MdJAZ10 (F). Immunoblotting with a GST antibody showing that MdJAZ5 and MdJAZ10 were pulled down by MdWRKY9-HIS.

To assess whether other proteins exhibit synergies with MdWRKY9 in response to salt stress, we used MdWRKY9 without its self-activating domain (MdWRKY9-F5) [44] obtained earlier as bait to screen for potential interacting proteins. We identified 26 positive clones, and interestingly, among them were two proteins belonging to the JAZ family (Table S2). In the JA-signaling pathway, JASMONATE-ZIM DOMAIN (JAZ) proteins typically act as repressors and can interact with other TFs to mediate various stress processes [45]. To verify the interaction between MdWRKY9 and MdJAZ5/MdJA10, direct Yeast Two-Hybrid (Y2H) assays were performed. The results indicated that yeast cells cotransformed with MdWRKY9-F5 and MdJAZ5/MdJAZ10 grew normally on SD selective medium −L/−T/−H/−A, indicating an interaction between MdWRKY9 and MdJAZ5/MdJAZ10 (Fig. 4A).

We then used luciferase complementation imaging (LCI) assays to test the interaction between MdWRKY9 and MdJAZ5/MdJAZ10. Tobacco (*N. benthamiana*) leaves coinjected with MdWRKY9-cLUC and MdJAZ5/MdJAZ10-nLUC produced a stronger luminescence signal compared to the empty vector control (Fig. 4B and C), demonstrating their interaction *in vivo*. The interaction was also confirmed using a bimolecular fluorescence complementation (BiFC) assay in onion epidermal cells. When MdWRKY9 and MdJAZ5/MdJAZ10 were coexpressed, strong yellow fluorescent protein (YFP) signals were detected in the nucleus (Fig. 4D). Furthermore, we also used a pull-down assay to test the *in vitro* interaction between MdWRKY9 and MdJAZ5/MdJAZ10. The results showed that the reconstructed glutathione-S-transferase (GST) fusion proteins MdJAZ5-GST and MdJAZ10-GST were both pulled down by MdWRKY9-HIS (Fig. 4E and F), further indicating an interaction between them.

### MdJAZ5 and MdJAZ10 inhibit the DNA-binding and transcriptional activity of MdWRKY9

**Figure 5:**
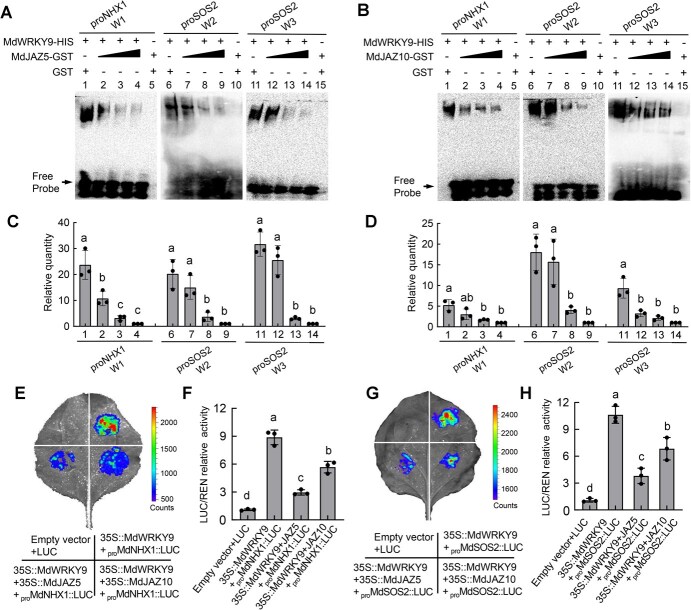
MdJAZ5 and MdJAZ10 inhibit the DNA-binding and transcriptional activity of MdWRKY9. (A–B) EMSA results indicate a marked suppression in the binding affinity of MdWRKY9 to the promoters of *MdNHX1* and *MdSOS2* upon the introduction of MdJAZ5 (A) and MdJAZ10 (B), respectively. To ensure consistent protein content across all samples, an empty GST protein was included as a control. (C–D) The probe-bound proteins undergo a relative quantitative analysis. (E–F) Transient LUC reporter assays showing the transcriptional activation of MdWRKY9 to the *MdNHX1* promoters with addition of MdJAZ5 and MdJAZ10. (G–H) Transient LUC reporter assays showing the transcriptional activation of MdWRKY9 to the *MdSOS2* promoters with addition of MdJAZ5 and MdJAZ10.

Since the protein repressors MdJAZ5 and MdJAZ10 can both interact with MdWRKY9 to form a complex, we next examined the effects of the JAZ–WRKY complex on the DNA-binding and transcriptional activities of MdWRKY9. To validate DNA-binding activity, we performed EMSA assays using both MdJAZ5-GST, MdJAZ10-GST and MdWRKY9-HIS fusion proteins. Empty GST protein alone was used to standardize the total protein amount in each tube. The binding strength of MdWRKY9 to the W-boxes of the *MdNHX1* and *MdSOS2* promoters was significantly inhibited by the addition of increasing concentrations of both MdJAZ5-GST and MdJAZ10-GST proteins (Fig. 5A–D), indicating that MdJAZ5 and MdJAZ10 suppress the binding activity of MdWRKY9. Moreover, quantitative analysis of the binding bands indicates that MdJAZ5 appears to have a stronger inhibitory effect on MdWRKY9 than MdJAZ10 (Fig. 5C and D).

On the other hand, to verify the effect of the JAZ–WRKY complex on the transcriptional activity of MdWRKY9, we further conducted transient LUC assays in *N. benthamiana* leaves. The CDS of *MdJAZ5* and *MdJAZ10* were also fused with the pGreenII 62-SK vector as coeffectors. Luminescence detection showed that when MdJAZ5 or MdJAZ10 were added as coeffectors, the luminescence intensity of the coexpression of 35S::MdWRKY9 + 35S::MdJAZ5 or 35S::MdJAZ10 with *proMdNHX1::LUC* was significantly lower than that of the expression of 35S::MdWRKY9 with *proMdNHX1::LUC* alone (Fig. 5E and F). The same results were also obtained in the detection of *proMdSOS2::LUC* promoter activity (Fig. 5G and H), indicating that the transcriptional activation activity of MdWRKY9 was suppressed by MdJAZ5 and MdJAZ10. Additionally, consistent with the trend of inhibiting binding activity, the inhibitory effect of MdJAZ5 on the transcriptional activation activity of MdWRKY9 is also stronger than that of MdJAZ10 (Fig. 5F and H). The above results suggest that MdJAZ5 and MdJAZ10, by directly interacting with MdWRKY9, inhibit its DNA-binding activity and suppress its transcriptional activation of downstream target genes.

### Exogenous MeJA treatment degrades JAZ proteins and releases transcriptional activation of MdWRKY9

**Figure 6:**
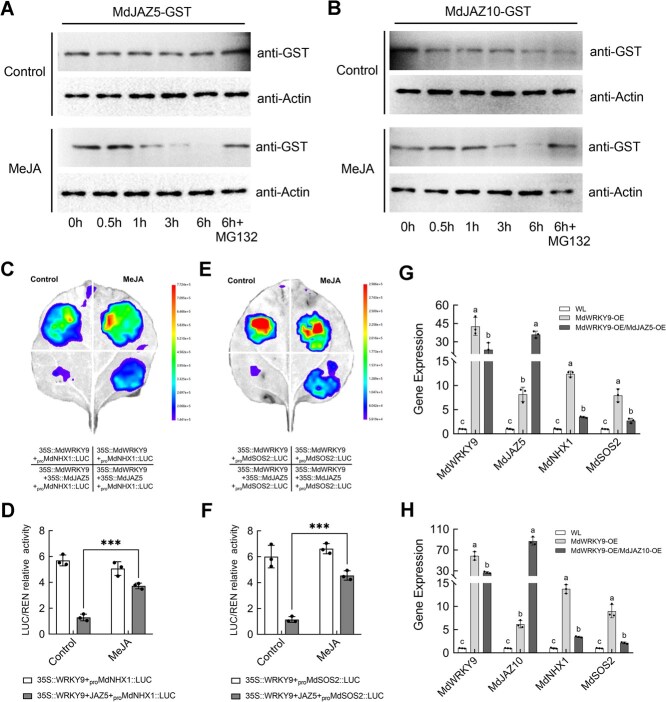
Exogenous MeJA treatment degrades JAZ proteins and releases transcriptional activation of MdWRKY9. (A–B) WT ‘Orin’ callus was subjected to either DMSO as a solvent control or 100 μM of MeJA treatment. Following treatment, extracts from these callus samples were combined with fusion proteins consisting of MdJAZ5-GST (A) and MdJAZ10-GST (B), respectively. These mixtures were then incubated for specified durations. To ensure equal protein loading across samples, actin served as a normalizing control. The degradation of JAZ proteins was inhibited by the MG132 treatment. (C–D) Transient LUC reporter assays showed that the transcriptional activation of MdWRKY9 to the *MdNHX1* promoter was significantly inhibited by MdJAZ5. This inhibitory effect can be significantly alleviated under exogenous MeJA treatment compared to the control. (E–F) Transient LUC reporter assays showed that the transcriptional activation of MdWRKY9 to the *MdSOS2* promoter was significantly inhibited by MdJAZ5. This inhibitory effect can be significantly alleviated under exogenous MeJA treatment compared to the control. (G) Transform WT ‘Orin’ callus with 35S::MdWRKY9 and 35S::MdWRKY9 + 35S::MdJAZ5, and quantify the expression levels of *MdWRKY9*, *MdJAZ5*, *MdNHX1*, and *MdSOS2*. (H) Transform WT ‘Orin’ callus with 35S::MdWRKY9 and 35S::MdWRKY9 + 35S::MdJAZ10, and quantify the expression levels of *MdWRKY9*, *MdJAZ10*, *MdNHX1*, and *MdSOS2*.

When stress induces JA synthesis, JAZ proteins respond to the JA signal and interact with COI1 in the SCF^COI1^ complex. This interaction leads to their ubiquitination and degradation by the 26S proteasome, thereby releasing TFs and activating the transcription of JA-responsive genes [45, 46]. To investigate the post-translational regulation of MdJAZ5 and MdJAZ10, we conducted protein degradation experiments *in vitro*. The results showed that both MdJAZ5-GST and MdJAZ10-GST fusion proteins were degraded more rapidly compared to the control when exogenous methyl jasmonate (MeJA) was added (Fig. 6A and B). However, when MG132 (a proteasome inhibitor) was added, the MeJA-induced degradation of MdJAZ5-GST and MdJAZ10-GST fusion proteins was significantly alleviated (Fig. 6A and B), indicating that MeJA can induce the degradation of MdJAZ5 and MdJAZ10 via the 26S proteasome. Compared with MdJAZ10, MdJAZ5 is more sensitive to MeJA treatment and is significantly degraded after 1 h of treatment (Fig. 6A).

Therefore, we further verified through transient LUC assays whether exogenous JA treatment could alleviate the inhibitory effect on the transcriptional activation activity of MdWRKY9 by degrading JAZ proteins. The results showed that the luminescence intensity of the coexpression of 35S::MdWRKY9 + 35S::MdJAZ5 with *proMdNHX1::LUC* was significantly lower than that of 35S::MdWRKY9 with *proMdNHX1::LUC* alone. However, this inhibitory effect caused by MdJAZ5 was significantly alleviated under exogenous MeJA treatment compared to the control (Fig. 6C and D). The same results were also obtained in the detection of *proMdSOS2::LUC* promoter activity (Fig. 6E and F), indicating that MeJA treatment promoted the degradation of MdJAZ5 and thus released the transcriptional activation activity of MdWRKY9. Similarly, the inhibitory effect of MdJAZ10 on the transcriptional activity of MdWRKY9 was also alleviated by exogenous MeJA treatment (Fig. S7A–D).

The response mechanism of the interaction between MdWRKY9 and MdJAZ5/MdJAZ10 under salt stress was explored through Agrobacterium-mediated transient transformation experiments. Using a CaMV 35S promoter as the driving element, we successfully cointroduced MdJAZ5 and MdJAZ10, respectively, with MdWRKY9 into the callus of ‘Orin’. Through RT-qPCR analysis, we confirmed the effective transformation and expression of *MdWRKY9*, *MdJAZ5*, and *MdJAZ10* genes in the callus tissue. Compared with the control group, the expression levels of *MdNHX1* and *MdSOS2* were significantly upregulated when MdWRKY9 was overexpressed alone. However, when MdWRKY9 was coexpressed with MdJAZ5 or MdJAZ10, although the expression levels of *MdNHX1* and *MdSOS2* remained higher than those in WT tissue, they were significantly reduced compared to the case where MdWRKY9 was overexpressed alone (Fig. 6G and H). The same results were obtained in the cotransformation experiment of tissue culture seedling GL3 (Fig. S8A and B).

The above results indicate that exogenous MeJA affects the stability of MdJAZ5 and MdJAZ10 proteins, which are subsequently degraded by the 26S proteasome, disrupting the JAZ–WRKY protein complex and thereby releasing MdWRKY9 to activate downstream gene expression.

## Discussion

Through long-term evolution, plants have developed a series of growth and development mechanisms to adapt to salt stress, among which hormone regulation is one of the core mechanisms [21]. JA is a lipid hormone endogenously synthesized by plants and plays an important role in regulating plant response to stress [47]. The application of exogenous JA enhances the antioxidant response in plants, thereby mitigating salt stress [48]. Research has demonstrated that the foliar application of MeJA significantly elevates the content of antioxidants such as anthocyanins in strawberries, bolstering their resistance to ROS accumulation induced by abiotic stresses [49]. In peas, MeJA treatment augments relative water content (RWC) and protein levels, thereby enhancing salt tolerance [50]. Similarly, in *Brassica napus*, the application of MeJA notably increases RWC, soluble sugar content, and photosynthetic rate, while reducing the damage inflicted by salt stress [51]. WRKY TFs play pivotal roles in stress response mechanisms. Studies have shown that GhWRKY25, GhWRKY939–1, and GhWRKY6 are crucial for cotton’s resistance to salt stress [52–54]. In apples, MdWRKY30 plays a significant role in conferring tolerance to osmotic and salt stresses [55]. Here, we found that salt stress significantly induced the accumulation of JA and the expression of a WRKY family TF, *MdWRKY9*, in apple roots. Transgenic callus overexpressing MdWRKY9 showed significantly higher growth and greater salt tolerance compared to WT ‘Orin’ callus. Transgenic *Arabidopsis* seedlings overexpressing MdWRKY9 also exhibited better growth, with significantly longer average root lengths compared to the WT control Col-0 (Fig. 2). In earlier studies, it was found that salt stress can trigger the activation of the jasmonate signaling pathway [28, 56]. The accumulation of endogenous JA levels in salt-tolerant crop varieties is significantly different from that in salt-sensitive varieties [57, 58]. These results all indicate the important role of JA in plant responses to salt stress, but the role of WRKY TFs and their regulatory mechanisms in this process still require further investigation.

WRKY TFs drive or inhibit the transcription of downstream target genes by binding to W-box elements in their promoters. In doing so, they participate in multiple salt stress response pathways, such as hormone signaling pathway, osmotic response pathway, oxidative stress response pathway, and SOS signaling pathway, acting as intermediaries in the interactions among different pathways [59, 60]. For example, in Arabidopsis, AtWRKY75 can bind to the *AtSOS1* promoter and activate its expression, while AtWRKY1 induces the expression of all three SOS genes (*AtSOS1*, *AtSOS2*, and *AtSOS3*) by binding to their promoters [61, 62]. In kumquat (*Fortunella crassifolia*), FcWRKY40 can bind to the *FcSOS2* promoter and activate its expression, thereby enhancing salt tolerance [63]. In apple, MdWRKY55 was reported to bind to the *MdNHX1* promoter and activate its expression, thereby regulating salt tolerance [38]. In the present study, we showed that overexpression of *MdWRKY9* can significantly induce the upregulation of *MdNHX1*and *MdSOS2* gene expression levels in the SOS signaling pathway (Fig. 2F and I). In this process, MdWRKY9 transcriptionally regulates *MdNHX1* and *MdSOS2* by binding to their promoters. MdWRKY9 specifically binds to one W-box (W1, −1255) in the *MdNHX1* promoter and to two W-boxes (W2, −149; W3, −303) in the *MdSOS2* promoter, thereby activating their transcription, as demonstrated by the EMSA and LUC reporter assays (Fig. 3). This is consistent with previous findings.

On the contrary, some WRKY TFs can negatively regulate plant salt tolerance by inhibiting the expression of genes in the SOS signaling pathway. For example, in sweet sorghum (*Sorghum bicolor* L. *Moench*), SbWRKY50 negatively regulates salt response by reducing the expression levels of the *Arabidopsis* Na^+^/H^+^ antiporter gene *AtSOS1* [37]. CmWRKY17 in chrysanthemum (*Chrysanthemum morifolium*) acts as a transcriptional repressor that negatively regulates the expression of salt stress-related genes *AtSOS1*, *AtSOS2*, *AtSOS3*, and *AtNHX1*, thereby inhibiting salt tolerance [64]. It is evident that different WRKY TFs exhibit varied functions in plant salt stress tolerance due to differences in the transcriptional regulation of downstream target genes. Our research findings indicate that WRKY9 can directly bind to the promoters of *MdNHX1* and *MdSOS2* to promote their transcriptional activity (Fig. 3). Intriguingly, the expression level of *MdSOS3* also increases to varying degrees in OE-MdWRKY9 calli. However, experimental evidence indicates that MdWRKY9 does not directly bind to the promoter of *MdSOS3* (Fig. 3B). The underlying regulatory mechanism by which MdWRKY9 indirectly elevates the transcriptional level of *MdSOS3* remains to be further investigated.

The JAZ proteins have been widely identified as transcriptional repressors in the JA signaling pathway, playing a crucial role in plant stress responses [45, 65]. Jasmonate treatment leads to the degradation of JAZ proteins, which depends on the activity of SCF^COI1^ ubiquitin ligase and the 26S proteasome, resulting in the activation of various TFs [45, 46]. It has been reported that TF families such as MYB, bHLH (MYC), and ERF are targets of JAZ proteins, which subsequently regulate the stress responses of their respective downstream pathways after their release [66, 67]. In this study, we used MdWRKY9 as the bait protein to screen for interacting proteins through a Y2H assay, identifying two JAZ proteins, MdJAZ5 and MdJAZ10. Several *in vitro* and *in vivo* interaction assays showed that MdWRKY9 interacts with both MdJAZ5 and MdJAZ10 (Fig. 4). Moreover, we found that the binding strength of MdWRKY9 to the W-boxes of the *MdNHX1* and *MdSOS2* promoters was significantly inhibited by its interaction with MdJAZ5 and MdJAZ10 (Fig. 5A–D). Transient LUC assays indicated that the transcriptional activation activity of MdWRKY9 on downstream genes was also inhibited by MdJAZ5 and MdJAZ10 (Fig. 5E–H). In recent studies, it was also found that WRKY TFs can interact with JAZ proteins and thus participate in the JA signal transduction pathway. In soybean (*Glycine max* L.), GmWRKY40 was reported to interact with eight JAZ proteins and thus participate in JA signaling-mediated resistance to *Phytophthora sojae* [68]. Furthermore, we further confirmed that JA signaling can indeed trigger the degradation of MdJAZ5 and MdJAZ10 proteins by the 26S proteasome, disrupting the JAZ–WRKY protein complex, and thereby releasing MdWRKY9 to activate downstream gene expression (Fig. 6), which is consistent with previous reports. Under salt stress, MdWRKY9 is released and subsequently activates the downstream SOS signaling pathway. Meanwhile, our research indicates that when plants are treated with JA under salt stress, the PRO content within the plants significantly increases (Fig. 1F). Whether MdWRKY9 is involved in the synthesis of PRO remains to be further investigated.

**Figure 7:**
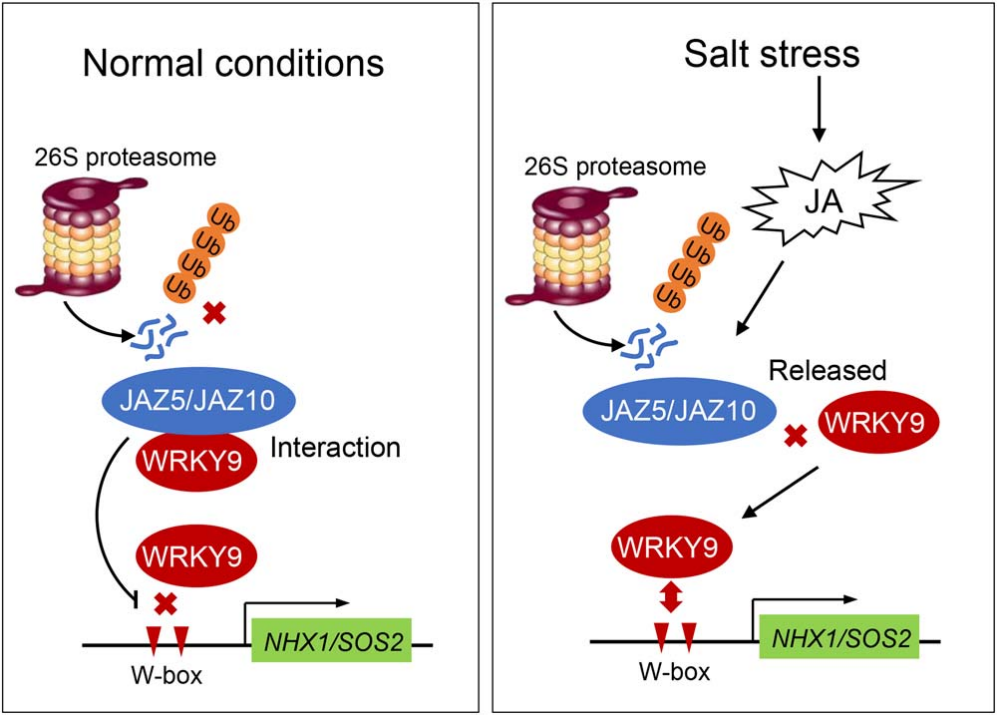
A proposed working model on the role of MdWRKY9 in JA-mediated salt tolerance in apple. Under normal conditions, MdJAZ5 and MdJAZ10 in the JA-signaling pathway can interact with MdWRKY9 to form a complex and inhibit its DNA-binding and transcriptional activation activity. However, under salt stress conditions, JA accumulation is induced, triggering the degradation of MdJAZ5 and MdJAZ10 proteins by the 26S proteasome. This disruption of the JAZ–WRKY protein complex releases MdWRKY9 to activate the expression of downstream genes *MdNHX1* and *MdSOS2*, thereby enhancing apple’s salt tolerance through ion homeostasis regulation.

In conclusion, based on the results of this study, we proposed a working model for the role of MdWRKY9 in JA-mediated salt tolerance in apple (Fig. 7). Under normal conditions, MdJAZ5 and MdJAZ10 in the JA signaling pathway can interact with MdWRKY9 to form a complex and inhibit its DNA-binding and transcriptional activation activity. However, under salt stress conditions, JA accumulation is induced, triggering the degradation of MdJAZ5 and MdJAZ10 proteins by the 26S proteasome. This disruption of the JAZ–WRKY protein complex releases MdWRKY9 to activate the expression of downstream genes *MdNHX1* and *MdSOS2*, thereby enhancing apple’s salt tolerance through ion homeostasis regulation.

## Materials and methods

### Plant materials and treatments

The apple seedlings (*M. hupehensis* Rehd.) were placed in the plant growth chamber, and the environment of the growth chamber was photoperiod 16 h, a temperature of 26°C ± 1°C and a humidity of 55% ± 5%. When the seedlings reached a height of 15 cm, seedlings with similar growth states were selected and subsequently irrigated with different concentrations of NaCl (0, 50, 100, 150, 200, and 250 mM). The M9T337 apple seedlings were subjected to identical environmental conditions, with irrigation of 150 mM NaCl every 3 days. Based on preliminary tests, 25 μM MeJA was identified as the optimal concentration for alleviating salt stress. The experimental group was sprayed with 25 μM MeJA every 3 days, while the control group was sprayed with an equivalent amount of ddH_2_O. This treatment lasted for 15 days.

The ‘Orin’ callus culture environment is carried out as described previously [69]. For salt treatment, ‘Orin’ callus and transgenic callus with the same growth state were selected and placed on MS medium with different salt concentrations (0, 100, and 200 mmol·L^−1^NaCl).

*Arabidopsis* seeds were soaked in 1 ml 70% ethanol for 15 s, rinsed with sterile ddH_2_O five times to remove residual ethanol, then soaked in 3% sodium hypochlorite for 10 min, rinsed eight times with sterile ddH2O to remove residual sodium hypochlorite, and finally seeded on one-half MS medium. After being vernalized at 4°C for 3 days, the seeds were subsequently cultured in a light incubator maintained at 22°C, subjected to a 16-h light period followed by an 8-h dark cycle. For salt treatment, *Arabidopsis* seeds were uniformly seeded on one-half MS medium containing 100 mmol·l^−1^ concentrations of NaCl.

### Detection of endogenous JA and JA-ile content

The determination method for JA involves using a Shimadzu LC-20A chromatograph equipped with a C18 column (250 × 4.6 mm, 5 μm) and a PDA diode array detector to analyze samples at a wavelength of 210 nm, with a flow rate of 1 ml/min, a column temperature of 35°C, and an injection volume of 10 μl of automatically sampled material. The mobile phase consists of acetonitrile (A) and 1% formic acid in water (B) at a constant ratio of A:B = 45:55. Prior to analysis, samples are homogenized with 1 ml of extraction solution, adjusted to a pH of 2.5–3.0 with 1 ml of 2 M HCl, transferred to an EP tube after mixing, extracted by ultrasound for 30 min, centrifuged at 8000 g, and the supernatant is collected. The residue is reextracted with 0.3 ml of extraction solution, and the supernatants from both extractions are combined, dried under nitrogen in an ice bath, reconstituted with 0.3 ml of the mobile phase, filtered, and then analyzed.

To determine JA-Ile, frozen biological samples are ground in liquid nitrogen using a low-temperature grinder. A precise amount of the ground sample is mixed with an internal standard solution and an extraction solvent (methanol/water/formic acid). The mixture is vortexed to homogeneity, then subjected to ultrasonic treatment and extraction at 4°C. After shaking, the sample is centrifuged, and the supernatant is collected. The residue is reextracted with methanol, and the supernatants are combined and concentrated. The concentrated sample is reconstituted with a methanol/water solution, filtered, and placed in a sample vial. Finally, the sample is analyzed using Ultra Performance Liquid Chromatography-Mass Spectrometry/Mass Spectrometry (UPLC-MS/MS) with specific chromatographic and mass spectrometry conditions.

### Physiological indexes

Fresh plant samples were rapidly frozen in liquid nitrogen, and the measurement procedures for PRO (Suzhou Keming Biotechnology Co., Ltd.) and MDA (Suzhou Keming Biotechnology Co., Ltd.) were carried out according to the instructions provided with the reagents.

### Genetic transformation

The full-length CDS of *MdWRKY9* without the stop codon was cloned into a CaMV 35S promoter-driven vector pRI101-AN with a GFP label. The full-length CDS of MdJAZ5 and MdJAZ10 without the stop codon was cloned into a CaMV 35S promoter-driven vector pPZP211–3 × Flag. The EHA105 strain of *Agrobacterium tumefaciens* was transformed with the recombinant vector.

After a 15- to 25-min infection, the apple callus was transferred to MS solid medium for dark culture for 48 h, and then the apple callus containing the recombinant pRI101-AN vector was transferred to kanamycin screening medium.

Apple tissue-cultured seedlings that have been grown for ~20 days and in good condition are selected. The Agrobacterium cells are suspended in ~30 ml of infiltration buffer (containing 10 mM MgCl_2_, 150 μM AS, and 10 mM MES). The leaves are then submerged into the infiltration solution and subjected to vacuum treatment for 40 min. Following this, the leaves are placed onto regeneration medium and incubated in the dark for 3 days.

As described previously, the transgenic *Arabidopsis* seedlings were generated [70]. The primers used in cloning are listed in Table S1.

### EMSA

The full-length CDS of *MdWRKY9* was cloned into the pET32a (+) vector fused with a His label, and the recombinant plasmid was introduced into *Escherichia coli* BL21(DE3) cells for protein expression and purification. EMSA flow was performed referring to the method previously reported as described previously [35].

### Transient LUC reporter assay

The *MdWRKY9*, *MdJAZ5*, and *MdJAZ10* CDSs were inserted into separate pGreenII 62-SK vectors, and the promoters of *MdNHX1* and *MdSOS2* were inserted into separate pGreenII 0800-LUC vectors [69]. These recombinant vectors were subsequently transferred into the *A. tumefaciens* GV3101 strain. Following the inoculation of tobacco plants, they were maintained in darkness for 36 h. Subsequently, each leaf was treated such that one-half received a 50-μM solution of MeJA while the other half was treated with water as a control. The leaves were then kept in the dark for an additional 3 h. Transient LUC reporter assay flow was performed referring to the method previously reported as described previously [35].

### ChIP-PCR assays

The GFP and MdWRKY9-GFP transgenic apple calli were immersed in fixation solution and then lysed using an ultrasonic disintegrator (JY92-IIDN; Scientz, Ningbo, China). The MdWRKY9 protein–DNA fragments were collected using GFP antibodies and A/G magnetic beads. Real-time fluorescent quantitative PCR was employed to detect the enrichment values of the *MdNHX1* and *MdSOS2* promoter fragments. The primers used for ChIP-PCR are listed in Table S1.

### Y2H assays

The sequence of *MdWRKY9-F5* without its self-activating domain obtained earlier [44] was recombined into the pGBKT7 bait vector. The pGADT7 vector was reassembled with the full-length CDS of *MdJAZ5* and *MdJAZ10*. Primers for gene cloning and vector construction are listed in Table S1. Y2H assays flow was performed referring to the method previously reported as described previously [35].

### Firefly LCI assays

The full-length CDS of *MdWRKY9*, *MdJAZ5*, and *MdJAZ10* were cloned into pCAMBIA1300-cLUC and pCAMBIA1300-nLUC, resulting in the constructs MdWRKY9-cLUC, MdJAZ5-nLUC, and MdJAZ10-nLUC. The subsequent experiment was carried out as described previously [44]. The primers used are listed in Table S1.

### BiFC assay

The CDS of *MdJAZ5* and *MdJAZ10* were cloned into the pSPYCE-35S vector, and the CDS of *MdWRKY9* was cloned into the pSPYNE-YFP vector. The primers used for cloning are listed in Table S1. BIFC flow was performed referring to the method previously reported as described previously [44].

### Pull-down assay

Full-length CDS of *MdWRKY9* was first cloned into the pET-32a (+) vector. *MdJAZ5* and *MdJAZ10* were cloned into pGEX-4 T-1, respectively, to obtain GST-tagged fusion proteins. Pull-down assay flow was performed referring to the method previously reported as described previously [44]. Primer details are listed in Table S1.

### *In vitro* protein degradation assay

The *in vitro* protein degradation assay was conducted as previously described [71]. The WT ‘Orin’ callus was treated with 100 mM MeJA, 100 mM MG132, and DMSO (inhibitor), and the extraction solution was added after grinding with liquid nitrogen. Extract on ice for 15 min. For the indicated times, the extract underwent separate incubations with the MdJAZ5-GST and the MdJAZ10-GST fusion proteins. Using anti-GST monoclonal antibodies, the relative abundance levels of MdJAZ5 and MdJAZ10 were detected via western blot analysis.

## Accession Numbers

The following accession numbers in the National Center for Biotechnology Information databases can be used to locate the sequence data from this article: MdWRKY9(LOC103433719), MdJAZ5(LOC103443669), MdJAZ10(LOC103417085), MdNHX1(LOC103423578), MdNHX2(LOC103416793), MdSOS1(LOC103403471), MdSOS2(LOC103448071), MdSOS3(LOC103439871), and MdActin(LOC114819919).

## Supplementary Material

Web_Material_uhaf068

## Data Availability

We confirm that data supporting the results are available in the present article and its Supplementary Information. RNA-seq data that support the findings of this study have been deposited in the NCBI Bioproject database under accession number PRJNA1047940.
