# Supplementary material for: The transcription factor MdWRKY9 is involved in jasmonic acid-mediated salt stress tolerance in apple
Source: Hortic Res. 2025 Mar 4;12(6):uhaf068. doi: 10.1093/hr/uhaf068 (PMC12038257; doi:10.1093/hr/uhaf068)
Supplement: Web_Material_uhaf068 [file web_material_uhaf068.zip › Supplemental Figure S1-S8.pdf]

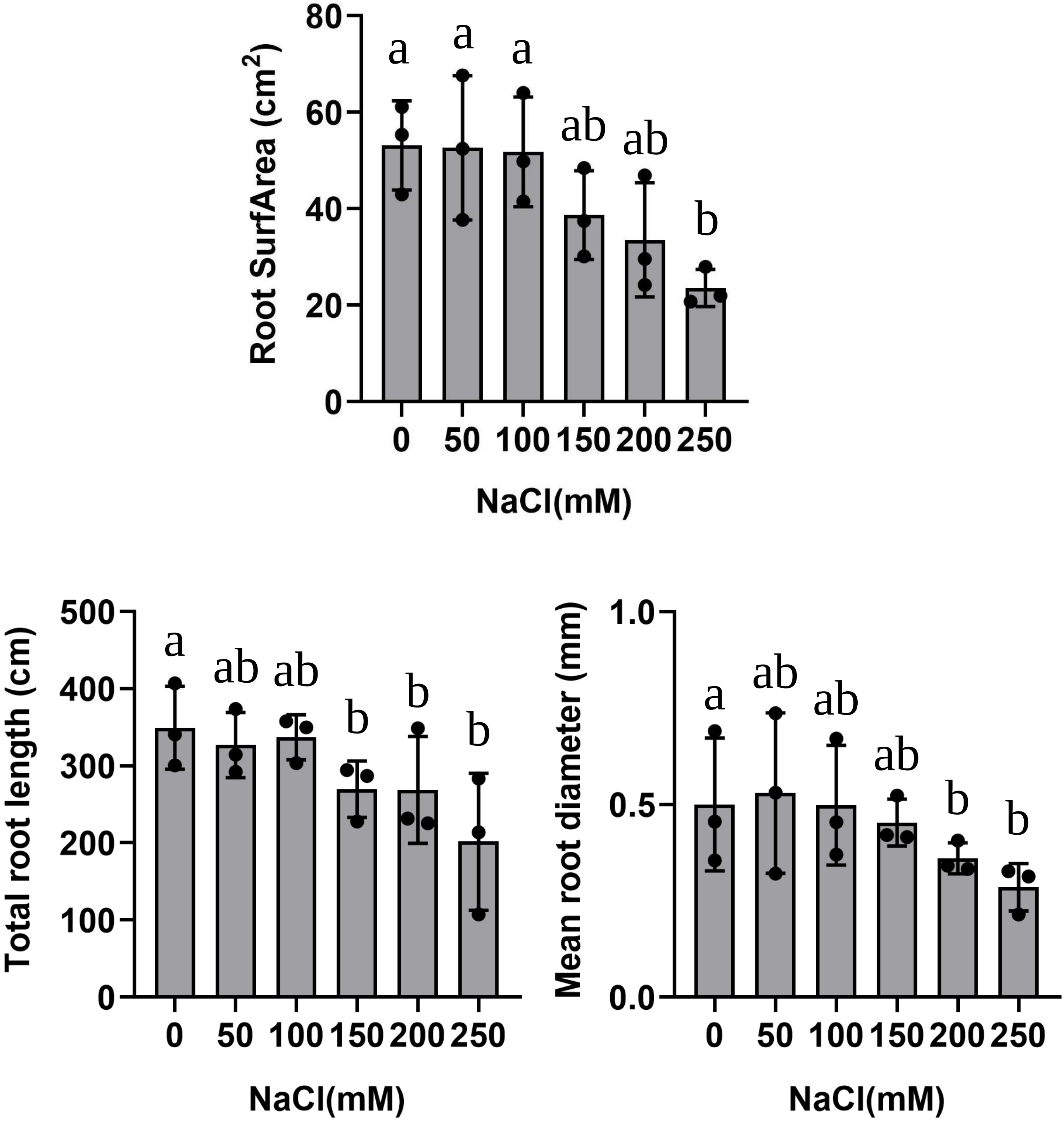

Supplemental Figure S1. Root surface area, total root length and mean root diameter of apple seedlings after salt treatment with different NaCl concentrations (0, 50, 100, 150, 200 and 250 mM). Values are means ± SD of three independent biological replicates (*n* = 3). Significant differences were determined by one-way ANOVA followed by a Tukey's test ($P < 0.05$).

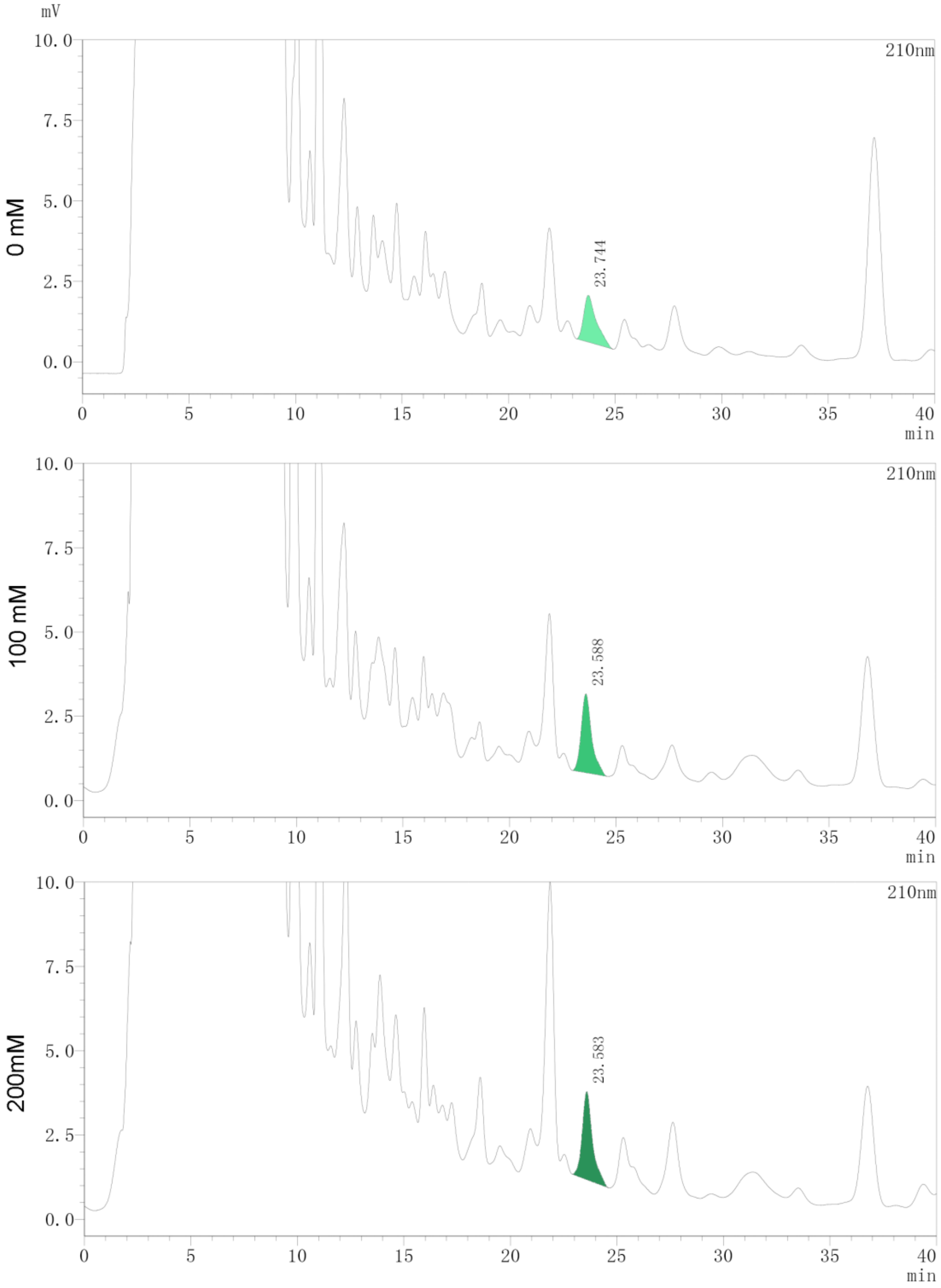

Supplemental Figure S2. Chromatograms of endogenous JA content in apple roots at NaCl concentrations of 0 mM, 100 mM, and 200 mM. The green part represents the peak area of JA.

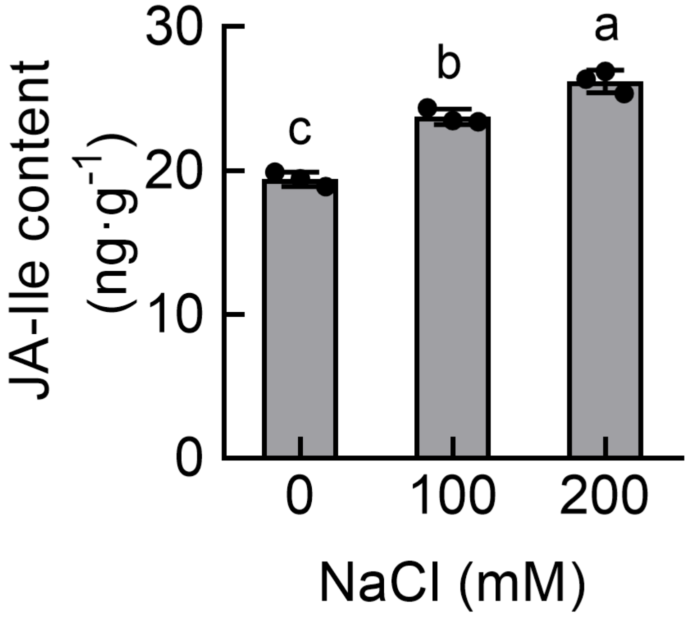

Supplemental Figure S3. Endogenous JA-Ile content accumulated in apple roots at NaCl concentrations of 0 mM,100 mM, and 200 mM. Values are means ± SD of three independent biological replicates (n = 3). Significant differences were determined by one-way ANOVA followed by a Tukey's test ($P < 0.05$).

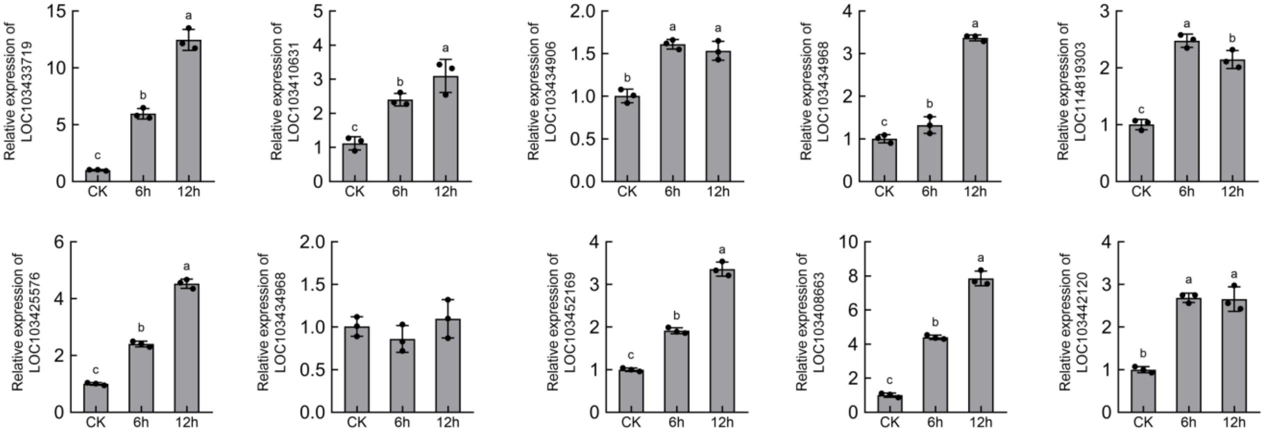

Supplemental Figure S4. Validate the differentially expressed WRKY transcription factors in the root system of M9T337 subjected to salt stress for 6 hours and 12 hours by RT-qPCR.

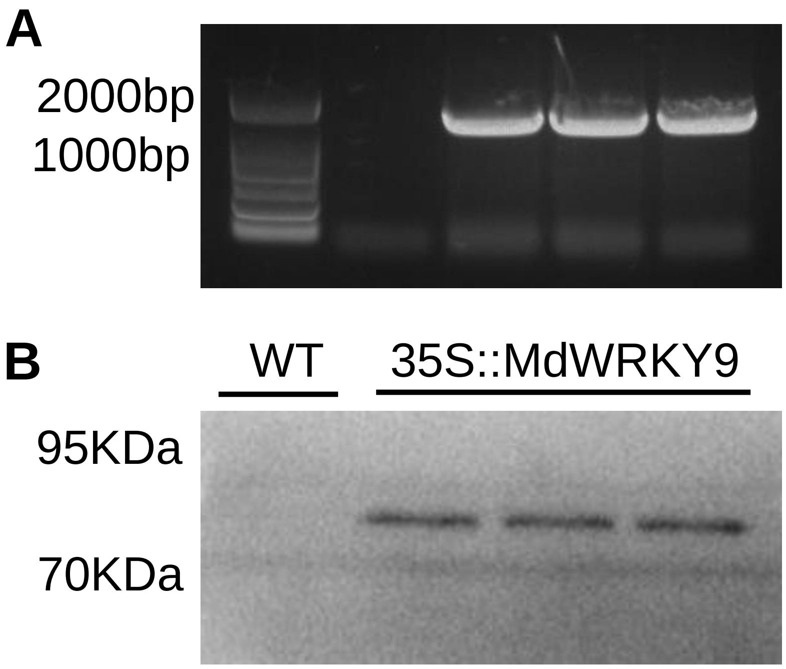

Supplemental Figure S5. Transgenic callus with overexpressed *MdWRKY9* (35S::MdWRKY9) were identified using (A) PCR and (B) immunoblotting.

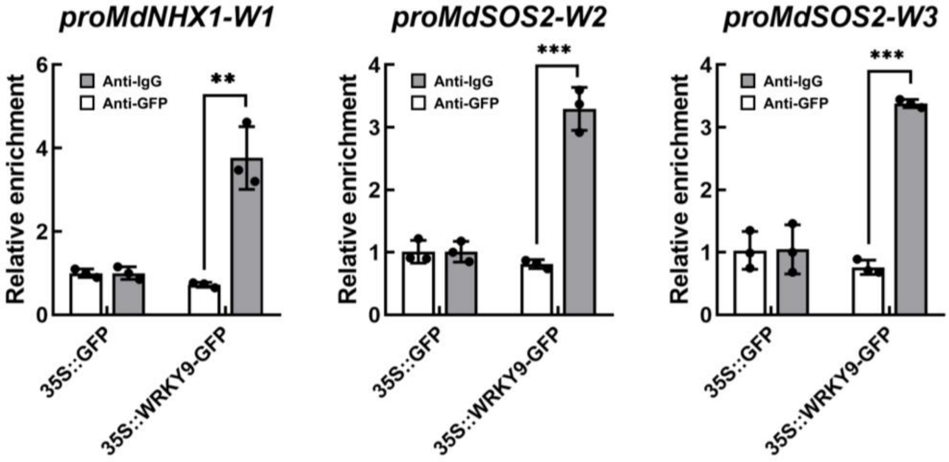

Supplemental Figure S6. The ChIP-qPCR assay demonstrated the binding of MdWRKY9 to the candidate W-box elements within the promoters of *MdNHX1* and *MdSOS2 in vivo*. DNA fragments enriched through ChIP were utilized as templates for qPCR. Significant differences were determined by one-way ANOVA followed by a Tukey's test (**$P < 0.01$, ***$P < 0.001$)

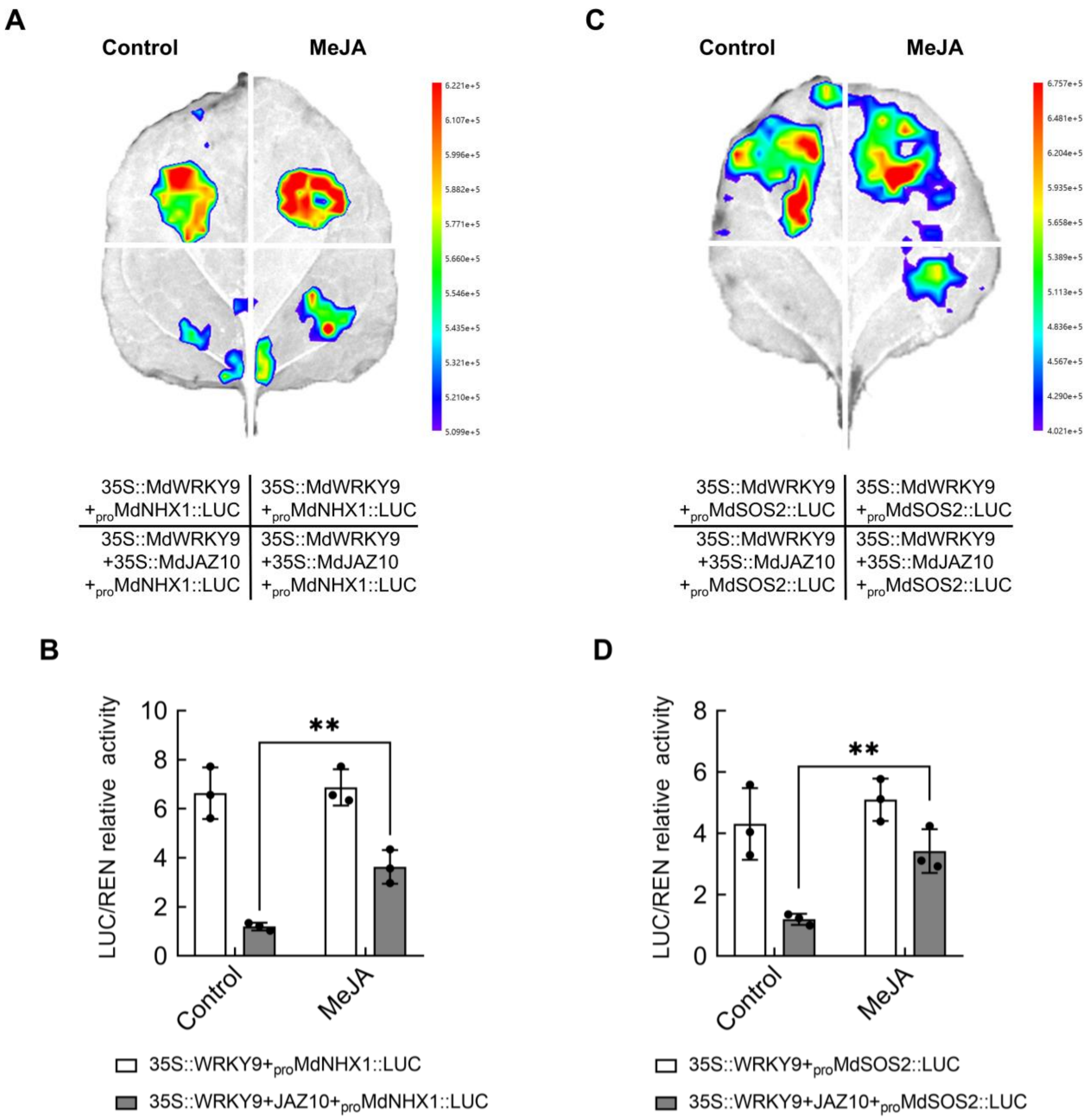

Supplemental Figure S7. (A–B) Transient LUC reporter assays showed that the transcriptional activation of MdWRKY9 to the *MdNHX1* promoter was significantly inhibited by MdJAZ10. This inhibitory effect can be significantly alleviated under exogenous MeJA treatment compared to the control. (C–D) Transient LUC reporter assays showed that the transcriptional activation of MdWRKY9 to the *MdSOS2* promoter was significantly inhibited by MdJAZ10. This inhibitory effect can be significantly alleviated under exogenous MeJA treatment compared to the control. Values are means ± SD of three independent biological replicates (n = 3). Significant differences were determined by one-way ANOVA followed by a Tukey's test (**P < 0.01, *P < 0.05).

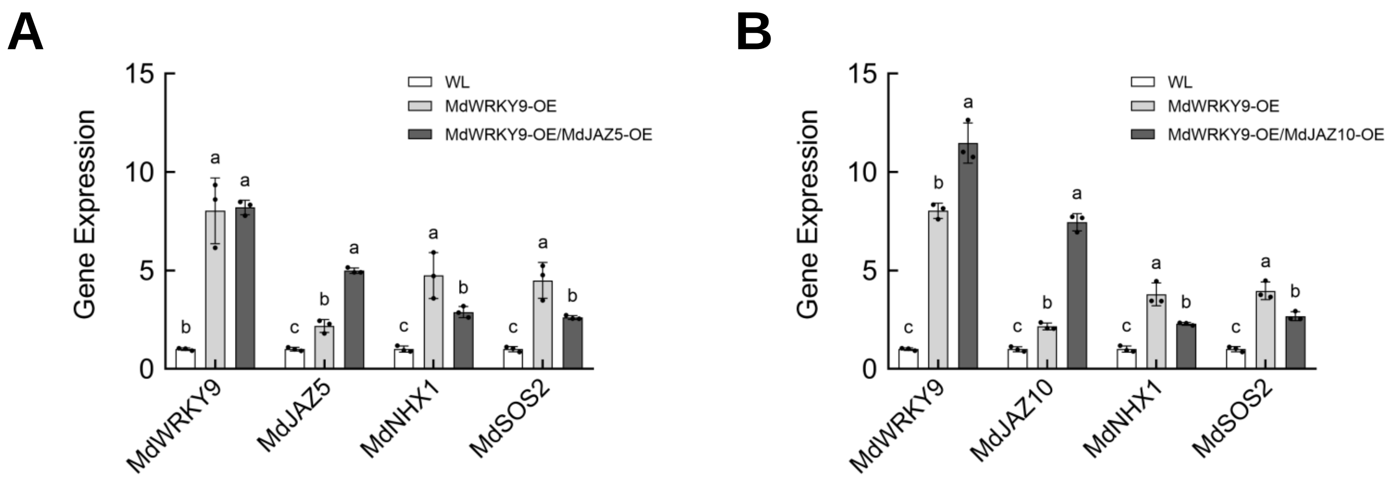

Supplemental Figure S8. (A) Transform GL3 tissue-cultured seedlings with 35S::MdWRKY9 and 35S::MdWRKY9 + 35S::MdJAZ5, and quantify the expression levels of *MdWRKY9*, *MdJAZ5*, *MdNHX1*, and *MdSOS2*. (H) Transform GL3 tissue-cultured seedlings with 35S::MdWRKY9 and 35S::MdWRKY9 + 35S::MdJAZ10, and quantify the expression levels of *MdWRKY9*, *MdJAZ10*, *MdNHX1*, and *MdSOS2*.
